# Oncostatin M Maintains Naïve Pluripotency of mESCs by Tetraploid Embryo Complementation (TEC) Assay

**DOI:** 10.3389/fcell.2021.675411

**Published:** 2021-05-26

**Authors:** Xiaoying Ye, Chenglei Tian, Linlin Liu, Guofeng Feng, Kairang Jin, Haiying Wang, Jiyu Chen, Lin Liu

**Affiliations:** ^1^State Key Laboratory of Medicinal Chemical Biology, Nankai University, Tianjin, China; ^2^Department of Cell Biology and Genetics, College of Life Sciences, Nankai University, Tianjin, China; ^3^Novo Nordisk Foundation Center for Stem Cell Biology (DanStem), University of Copenhagen, Copenhagen, Denmark

**Keywords:** oncostatin M (OSM), LIF, ESC, naïve pluripotency, TEC mice, telomere, 2C-genes, Stat3

## Abstract

It has been well established that leukemia inhibitory factor (LIF) is essential for maintaining naïve pluripotency of embryonic stem cells (ESCs). Oncostatin M (OSM) is a member of the IL-6 family of cytokines which share gp130 as a receptor subunit, and the OSM-gp130 complex can recruit either LIF receptor β or OSM receptor β. Here we show that OSM can completely replace LIF to maintain naïve pluripotency of ESCs. Mouse ESCs (mESCs) cultured in the presence of LIF or OSM not only express pluripotency genes at similar levels but also exhibit the same developmental pluripotency as evidenced by the generation of germline competent chimeras, supporting previous findings. Moreover, we demonstrate by tetraploid embryo complementation assay, the most stringent functional test of authentic pluripotency that mESCs cultured in OSM produce viable all-ESC pups. Furthermore, telomere length and telomerase activity, which are also crucial for unlimited self-renewal and genomic stability of mESCs, do not differ in mESCs cultured under OSM or LIF. The transcriptome of mESCs cultured in OSM overall is very similar to that of LIF, and OSM activates Stat3 signaling pathway, like LIF. Additionally, OSM upregulates pentose and glucuronate interconversion, ascorbate and aldarate metabolism, and steroid and retinol metabolic pathways. Although the significance of these pathways remains to be determined, our data shows that OSM can maintain naïve pluripotent stem cells in the absence of LIF.

## Introduction

Mouse embryonic stem cells (mESCs) derived from the inner cell mass of preimplantation embryos are known to be in a state of naïve pluripotency. This condition is underscored by the derivation of healthy adult mice if the cells are introduced into a tetraploid donor blastocyst (Nagy et al., 1993). Leukemia inhibitory factor (LIF) is typically added to the culture medium to inhibit autonomous differentiation of mESCs, mainly by activating the Jak/Stat3 pathway (Niwa et al., 1998; Raz et al., 1999). Novel condition 2i (inhibitors of Mek and Gsk3β signaling) medium reportedly could retain self-renewal and multilineage commitment of embryoid bodies independent of LIF and Stat3 (Ying et al., 2008). Addition of LIF to 2i medium (2i/L medium) was reported to elevate the development potential of mESCs by completed-ESC pups, although the pups failed to survive to adulthood due to irreversible global DNA hypomethylation and impaired telomere function for long-term culture (Choi et al., 2017; Yagi et al., 2017; Guo et al., 2018). More specifically, LIF has been shown to play an essential role in underpinning the naïve pluripotency of mESCs not only in conventional serum medium but also in chemically defined 2i medium. Thus far, however, there have been no reports of a complete replacement for LIF in mESC cultures.

Oncostatin M (OSM) is a member of the interleukin (IL)-6 family and was originally characterized for its ability to promote differentiation of histiocytic lymphoma cells (Zarling et al., 1986). Of note, OSM has been reported to be structurally and functionally related to LIF, sharing both the transducer gp130 and LIF receptor beta (LIFRβ) (Gearing and Bruce, 1992; Gearing et al., 1992). Notably, mESCs cultured in OSM were shown to resemble, in morphology and expression of pluripotency markers, to mESCs grown in LIF-supplemented medium (Rose et al., 1994). Moreover, OSM was found to retain mESCs in pluripotency, as revealed by the detection of chimeras that were competent for germline transmission (Nichols et al., 1994). However, it remains elusive whether OSM could completely substitute LIF in maintaining the mESCs in naïve pluripotency by tetraploid embryo complementation, which is considered the most stringent test for complete developmental potential (Eggan et al., 2002).

In the present study, we demonstrate that OSM could maintain mESCs in naïve pluripotency, as validated by the generation of healthy adult mice from mESCs cultured in OSM for up to 10 passages using the TEC method. mESCs in OSM-supplemented medium exhibited equivalent pluripotency and 2-cell gene expression levels, telomere length, telomerase activity, overall transcriptome profile, efficiency of germline competent chimera, and generation of TEC mice, compared with mESCs cultured in LIF. In addition to activating the Jak/Stat3 signaling pathway, OSM was also found to upregulate pentose and glucuronate interconversion, ascorbate and aldarate metabolism, and steroid and retinol metabolic pathways, which might be involved in the self-renewal and maintenance of naïve pluripotency. However, this needs further investigation.

## Materials and Methods

### Mice and Cell Culture

Mice were housed in the College Animal Facility and the use of mice for this research was approved by the Institutional Animal Care and Use Committee at Nankai University. Balb/c and ICR mice were purchased from Beijing Vital River Laboratory Animal Technology Co., Ltd.

The mouse ES cell line used in this study was derived from the C57BL/6 × 129S6 blastocyte based on the method described (Huang et al., 2008). The mESCs were cultured on mitomycin C-inactivated MEF feeder cells in serum and LIF based conventional ESC culture medium for five passages. Then the mESCs were transferred to different conditions including ESC basic medium (-LIF medium), ESC basic medium with 1,000 U/mL LIF (Millipore) (LIF medium), and ESC basic medium with OSM (GenScript) (OSM medium), for another 5 (P5) and 10 (P10) passages. ESC culture medium was changed daily and cells routinely passaged every 2 days. Count the cells during passaging for proliferation curve. The ESC basic medium consisted of knockout DMEM (Invitrogen), 20% ESC-quality FBS (Hyclone), 0.1 mM non-essential amino acids (Sigma), 0.1 mM β-mercaptoethanol (Invitrogen), 1 mM L-glutamine (Invitrogen), penicillin (50 U/mL) and streptomycin (50 U/mL) (Invitrogen).

### Immunofluorescence Microscopy

Mouse ESCs were fixed in 3.7% paraformaldehyde in PBS for 30 min at 4°C, washed once in PBS then permeabilized in 0.1% Triton X-100 in blocking solution (3% goat serum plus 0.1% BSA in PBS) for 30 min at room temperature, washed once with PBS, and left in blocking solution for 2 h. Cells were incubated overnight at 4°C with primary antibodies against Oct4 (sc5279, Santa Cruz), Nanog (A300-397A, Bethyl), SSEA-1 (MAB4301, Millipore). ESCs were washed three times (each for 15 min) with blocking solution, and incubated for 2 h with secondary antibodies at room temperature. Goat Anti-Mouse IgG (H + L) FITC (115-095-003, Jackson) and Goat Anti-Rabbit IgG (H + L) Alexa Fluor^®^ 594 (111-585-003, Jackson), diluted 1:200 with blocking solution, were used. Samples were washed and counterstained with 0.5 μg/mL DAPI in Vectashield mounting medium (Vector Laboratories). Fluorescence was detected and imaged using Confocal laser scanning microscope LSM710 (Carl Zeiss).

### Gene Expression Analysis by Real-Time qPCR

Total RNA of mESCs at P10 was extracted with RNeasy Mini Kit (Qiagen), according to manufacturer’s instructions. The cDNA was generated from 2 μg total RNA using M-MLV Reverse Transcriptase (Invitrogen). Real-time quantitative PCR (qPCR) reactions were set up in duplicate with the FS Universal SYBR Green Master (Roche) and run on an iCycler MyiQ2 Detection System (Bio-Rad). Each sample was repeated three times and normalized using Gapdh as the internal control. The amplification was performed for primary denaturation at 95°C for 10 min, then 40 cycles of denaturation at 95°C for 15 s, annealing and elongation at 58°C for 1 min, and the last cycle under 55–95°C for the dissociation curve. Relative quantitative evaluation of the target gene was determined by comparing the threshold cycles. The primers are listed in Supplementary Table 1.

### Western Blot

Mouse ESCs were collected and lysed in cell lysis buffer on ice for 30 min and then sonicated for 1 min at 60 of amplitude at 2 s intervals. After centrifugation at 10,000 *g* at 4°C for 10 min, the supernatant was collected. The protein samples were boiled in SDS sample buffer at 95°C for 10 min then was resolved by 10% Acr-Bis SDS-PAGE and transferred to polyvinylidene difluoride membranes (PVDF, Millipore). Non-specific binding was blocked by incubation in 5% non-fat milk or 5% BSA solution for 2 h. Blots were then probed with primary antibodies overnight by incubation at 4°C with Oct4 (sc5279, Santa Cruz), Nanog (ab80892, Abcam), Lin28a (3978S, CST), Stat3 (ab76315, Abcam), pStat3 (#9131S, Cell Signaling), H3K4me3 (Abcam, ab213224), H3K27me3 (Abcam, ab177178), H3 (Abcam, ab1791), or β-Actin (P30002, Abmart) served as the loading control. Secondary antibodies HRP conjugated goat anti-rabbit IgG (NA934V, GE Healthcare) or goat anti-mouse IgG (H + L) (ZB2305, ZSGB-BIO) were used for incubation at room temperature for 2 h. Protein bands were visualized using Chemiluminescent HRP substrate (WBKLS0500, Millipore).

### FACS Analysis

For the FACS analysis of Zscan4 expression profile, mESCs at P10 were collected and washed with cold PBS, then fixed in cold 70% ethanol, permeabilized in 0.1% Triton X-100 in blocking solution (3% goat serum in PBS) for 30 min, washed three times, and left in blocking solution for 1 h. ESCs were incubated with primary antibody against Zscan4 (AB4340, Millipore) for 1.5 h, washed three times, and incubated for 1 h with secondary antibody Alex a Fluor 488 Goat Anti-Rabbit IgG (H + L) (A11008, Life) diluted 1:200 with blocking solution. Samples were washed three times with PBS and FACS analysis was performed using a Flow Cytometer (BD Biosciences).

### Telomere Measurement by Q-FISH

Telomere length was estimated by telomere Q-FISH as described previously (Herrera et al., 1999; Huang et al., 2011). Telomeres were denatured at 80°C for 3 min and hybridized with Cy3-labeled (CCCTAA)_3_ peptide nucleic acid (PNA) probe at 0.5 μg/mL (F1002, Panagene). Chromosomes were stained with 0.5 μg/mL DAPI. Fluorescence from chromosomes and telomeres were digitally imaged on a Zeiss microscope with Cy3/DAPI using AxioCam and AxioVision software 4.6. Telomere length showed as telomere fluorescence intensity was integrated using the TFL-TELO program (a gift kindly provided by P. Lansdorp).

### Telomerase Activity by TRAP Assay

Telomerase activity was determined by the Stretch PCR method according to manufacturer’s instruction using the TeloChaser Telome-rase assay kit (T0001, MD Biotechnology). About 2.5 × 10^4^ mESCs at P10 from each sample were lysed. Lysis buffer served as negative controls. PCR products of cell lysate were separated on non-denaturing TBE-based 12% polyacrylamide gel electrophoresis and visualized by ethidium bromide (EB) staining.

### Telomerase Assay by ELISA Assay

Telomerase level was determined by ELISA method according to the manufacturer’s instruction using Mouse Telomerase (TE) ELISA kit (CSB-E08022m, CUSABIO).

### Chimera Generation, Tetraploid Complementation, and Genotyping

To produce chimeric mice, 10–15 mESCs were injected into four or eight-cell embryos collected from Balb/c mice, using a piezo-actuated microinjection pipette. Injected embryos were cultured overnight in KSOM medium. Blastocysts were transplanted into the uterus of 2.5 dpc pseudo-pregnant ICR mice. For tetraploid embryo complementation assay, tetraploid embryos were first produced by electrofusion of two-cell stage embryos collected from ICR mice. Approximate 15 ESCs at P10 were subsequently injected into the cavity of the tetraploid blastocysts. The tetraploid complemented embryos were transplanted into the uterus of pseudo-pregnant ICR mice. Surrogate mother delivered pups naturally on approximately day 17.5 of gestation. DNA microsatellite genotyping analysis was performed using D12Mit136 and D8Mit4. The PCR primer sequences (Supplementary Table 2) were obtained from the Mouse Genome Informatics website.

### Library Preparation and RNA-Sequencing

Library Preparation and RNA-Sequencing mRNA was purified from total RNA extracted from mESCs at P10 using poly-T oligo-attached magnetic beads. Fragmentation was carried out using divalent cations under elevated temperature in NEB Next First Strand Synthesis Reaction Buffer (5×). First strand cDNA was synthesized using random hexamer primer and M-MLV Reverse Transcriptase (RNase H). Second strand cDNA synthesis was subsequently performed using DNA Polymerase I and RNase H. Remaining overhangs were converted into blunt ends via exonuclease/polymerase activities. After adenylation of 30 ends of DNA fragments, NEB Next Adaptors with hairpin loop structure were ligated to prepare for hybridization. To select cDNA fragments of preferentially 150–200 bp in length, the library fragments were purified with AMPure XP system (Beckman Coulter, Beverly, United States). Then 3 mL USER Enzyme (NEB, United States) was used with size-selected and cDNA adaptor ligated at 37°C for 15 min followed by 5 min at 95°C prior to PCR. PCR was performed with Phusion High-Fidelity DNA polymerase, Universal PCR primers and Index Primer. At last, PCR products were purified using AMPure XP system and library quality assessed on the Agilent Bioanalyzer 2100 system. Cluster of the index-coded samples was performed on a cBot Cluster Generation System using TruSeq PE Cluster Kit (Illumina) according to the manufacturer’s instructions. After cluster generation, the library preparations were sequenced on an Illumina Hiseq platform.

### Bioinformatics Analysis

Clean reads were mapped to the mouse reference mm10 reference genome using Hisat2 (Kim et al., 2015). Reads were assigned and counted to genes using the Featurecounts (Liao et al., 2014). The read counts were then loaded into RStudio (R version 3.5), and DESeq2 was used to identify differentially expressed genes. Functional enrichments (GO annotation or KEGG) of differential genes were performed using clusterProfiler (Yu et al., 2012). The heat-maps were drawn by the function “pheatmap” of R packages, correlation coefficients were calculated by the function “cor” in R. Scatterplots were generated using the “ggplot2” package to graphically reveal genes that differ significantly between two groups. Corrected *P*-value of 0.05 and log_2_ (fold change) of 1 were set as the threshold for significantly differential gene expression.

### Quantification and Statistical Analysis

Statistics were analyzed using the GraphPad Prism. Data were analyzed using two-tailed unpaired Student’s *t*-test to compare two groups or ANOVA to compare more than two groups and expressed as Mean ± SEM. *P-*values less than 0.05 were considered significant (^∗^*P* < 0.05, ^∗∗^*P* < 0.01 or ^∗∗∗^*P* < 0.001). In addition, data of TFU is expressed as Mean ± SD in Figure 2B. FACS data were analyzed by FlowJo. TFU of telomere Q-FISH was quantified by TFL-TELO program. Graphs were generated using GraphPad Prism or R package ggplot2 and other R packages described in the method details.

## Results

### OSM Activates the Stat3 Pathway and Sustains Expression of Pluripotency Genes

It is known that LIF binds to the gp130/LIFRβ cell-surface receptor complex, which intracellularly bound to Jak1 to initiate activation of the Stat3 signaling cascade and the core pluripotency circuitry (Niwa et al., 2009). Activation of Stat3 is known to be critical for the maintenance of pluripotency in ESCs (Raz et al., 1999). We thus investigated whether the Stat3 pathway was activated by OSM and aimed to confirm the optimal concentration for this activation.

**FIGURE 1:**
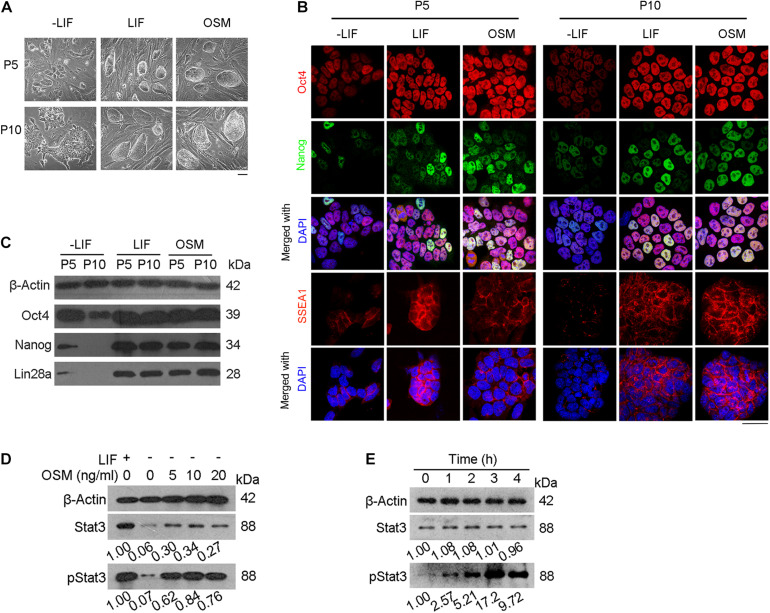
OSM activates Stat3 pathway and sustains expression of pluripotency genes. **(A)** Morphology of mESCs cultured in ESC basic medium (-LIF), ESC basic medium with LIF (LIF) and ESC basic medium with OSM (OSM). Scale bar, 100 μm. **(B,C)** Expression of pluripotency genes cultured in -LIF, LIF and OSM medium at P5 and P10 by immunofluorescence **(B)** and western blot **(C)**. Scale bar, 20 μm. **(D)** Western blot analysis of Stat3 activated by treatment with different concentrations of OSM for 24 h. **(E)** Western blot analysis of Stat3 activated by treatment with 10 ng/mL OSM for different lengths of time.

We used naïve mESCs at passage 5 in these experiments. Using western blot analysis, we measured the protein levels of Stat3 and pStat3 (phosphorylated Stat3) in the mESCs treated with different concentrations of OSM (5, 10, and 20 ng/mL) for 24 h. We also used mESCs cultured in LIF and LIF-free ESC culture medium as positive and negative controls, respectively. As expected, based on the shared receptors with LIF, we found that OSM activated Stat3 at concentrations of 5, 10, and 20 ng/mL. We also observed that phosphorylation of Stat3 reached its maximum level at 10 ng/mL OSM (Figure 1D). Based on this finding, we cultured mESCs in medium supplemented with 10 ng/mL OSM in all subsequent experiments. To determine the time required for OSM-triggered activation of Stat3, we treated mESCs with 10 ng/mL OSM for different periods (0, 1 2, 3, and 4 h). Our western blot analysis showed that after 1 h of incubation with OSM, the level of pStat3 increased, reaching its maximum level at 3 h, after which it decreased to a relatively stable level (Figure 1E).

Mouse ESCs were then cultured for another 5 (P5) and 10 (P10) passages in the ESC culture medium supplemented with 10 ng/mL OSM (OSM-ESCs) or 1,000 units/mL LIF (LIF-ESCs), with mESCs cultured in the absence of either LIF or OSM (-LIF-ESCs) serving as the control. We observed that morphologically, both LIF and OSM could sustain the undifferentiated cell state (characterized by compact domed cell colonies). In contrast, only differentiated clones (characterized by large, flat cells) could be observed in the -LIF-ESCs at the same passages (Figure 1A). Furthermore, immunofluorescent staining indicated that the expression of pluripotency-associated proteins (Oct4, Nanog, SSEA1) did not differ in mESCs cultured in either OSM or LIF at either P5 or P10, whereas they were dramatically decreased in -LIF-ESCs, suggesting that these cells underwent dramatic cellular differentiation (Figure 1B). This result was further validated by western blot analysis for Oct4, Nanog, and Lin28 (Figure 1C).

### OSM Facilitates Normal Telomere Function and Heterogeneity in 2-Cell Gene Expression

**FIGURE 2:**
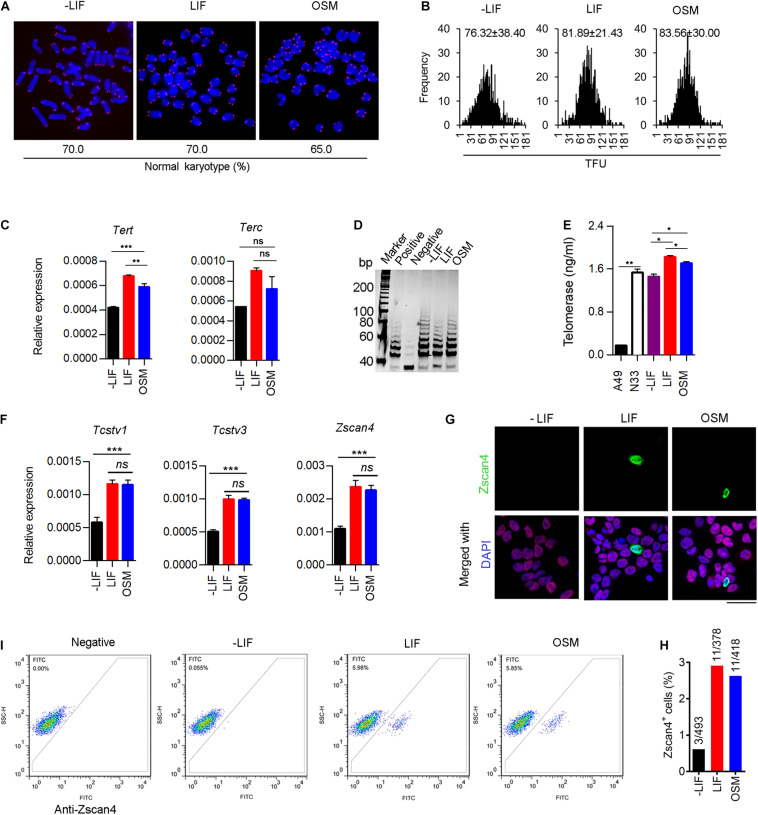
OSM maintains telomere elongation, telomerase activity and heterogeneity in 2-cell gene expression. **(A)** Representative images displaying telomere Q-FISH of mESCs cultured in -LIF, LIF, and OSM medium. Blue, chromosomes stained with DAPI; Red dots, telomeres. Bottom panel, the ratio of normal karyotype in the three culture conditions. **(B)** Telomere Q-FISH assay showing telomere length distribution by telomere fluorescence unit (TFU). Data shown as Mean ± SD. **(C)** Expression of telomerase-related genes: *Tert* and *Terc* in mESCs cultured in -LIF, LIF and OSM medium. **p < 0.01; ***p < 0.001; ns, no significant difference. **(D)** Telomerase activity measured by TRAP assay. Lysis buffer served as negative control. **(E)** Telomerase activity measured by ELISA assay. A49 mESCs (*Terc*^–/–^ G4 ESCs) and N33 mESCs (wild type ESCs) served as negative control and positive control, respectively. *p < 0.05; **p < 0.01. **(F)** Relative expression of 2-cell genes including *Tcstv1*, *Tcstv3*, and *Zscan4* in ESCs cultured in -LIF, LIF, and OSM medium. ***p < 0.001; ns, no significant difference. **(G)** Immunofluorescence staining of Zscan4 (green) and Oct4 (red) in ESCs cultured in -LIF, LIF, and OSM medium. Blue, DAPI. Scale bar, 20 μm. **(H)** Percentage of Zscan4^+^ cells (number of mESCs counted in immunofluorescence staining assay). **(I)** Flow cytometry diagram indicating percentage of Zscan4^+^ cells in mESCs cultured in -LIF, LIF, and OSM medium. Samples lacking the antibody served as a negative control.

Mammalian telomeres consist of TTAGGG_n_ repeat sequences at the end of chromosomes that are known to protect genomic stability, with the telomere length being maintained primarily by the action of telomerase (Blackburn et al., 2015; Liu, 2017). Telomere lengths have been highly correlated with the developmental pluripotency of mESCs (Huang et al., 2011). Therefore, we measured telomere length of mESCs chromosomes by telomere quantitative fluorescence *in situ* hybridization (Q-FISH). We observed that mESCs cultured under the three conditions described above exhibited normal karyotypes at P10 (Figure 2A). Moreover, telomere length (represented as telomere fluorescence intensity (TFU) in OSM-ESCs (83.56 ± 30.00 TFU) were similar to those of LIF-ESCs (81.89 ± 21.43 TFU). In contrast, we note that telomeres dramatically shortened in -LIF-ESCs (76.32 ± 38.40 TFU) over passages *in vitro* (Figure 2B), which is associated with the differentiating phenotype. Using quantitative real-time PCR (qRT-PCR), we found that the expression of *Tert* was significant at a higher level in OSM-ESCs than in -LIF-ESCs, though lower than in LIF-ESCs. There were no significant differences in the expression levels of *Terc* telomerase subunits in -LIF-ESCs, OSM-ESCs and LIF-ESCs (Figure 2C). To directly assess the telomerase activity, we performed both telomeric repeat amplification protocol (TRAP) and enzyme-linked aptamer sorbent assay (ELISA). The TRAP assay did not reveal any noticeable differences among -LIF-ESCs, LIF-ESCs, and OSM-ESCs in terms of their telomerase activity. For ELISA assay, we used the A49*Terc-*knockout ES cell line as the negative control, and the N33 wild-type ES cell line as the positive control. We observed that both OSM-ESCs and LIF-ESCs exhibited significant higher telomerase activity than -LIF-ESCs, whereas telomerase activity in OSM-ESCs was lower than in LIF-ESCs (Figures 2D,E). Together, these results indicated that OSM retained the length of telomeres and a relatively high telomerase activity to maintain the capacity of ESCs for indefinite self-renewal.

Naïve mESC cultures are known to be a heterogeneous mixture of metastable cells with fluctuating activation of 2-cell embryo specific genes (2C-genes), such as *Zscan4* (Falco et al., 2007), *Tcstv1/3* (Cerulo et al., 2014) and *MERVL* elements (Macfarlan et al., 2012). These 2C-like cells have been reported to exhibit an extended development ability, contributing to both embryonic and extraembryonic tissues, thus mimicking their *in vivo* counterparts, the totipotent 2-cell embryos. To determine whether OSM facilitates expression of 2C-genes, we analyzed the transcripts of *Zscan4*, *Tcstv1*, and *Tcstv3* by qRT-PCR. We did not observe any significant differences in the expression levels of these three genes between OSM-ESCs and LIF-ESCs. In contrast, their expression was dramatically decreased in ESCs grown without LIF or OSM (Figure 2F). Typically, mESC cultures (~5% of cell population) are known to express *Zscan4*, facilitating telomere elongation by telomere-sister chromatid exchange (T-SCE) (Zalzman et al., 2010). Immunofluorescence microscopy quantification and flow cytometry analysis confirmed the occurrence of a proper proportion of Zscan4-positive (Zscan4^+^) cells maintained in OSM-ESCs, similar to those in LIF-ESCs (Figures 2G–I).

### OSM Supports Efficient Production of Germline Transmission Mice and TEC Mice

**FIGURE 3:**
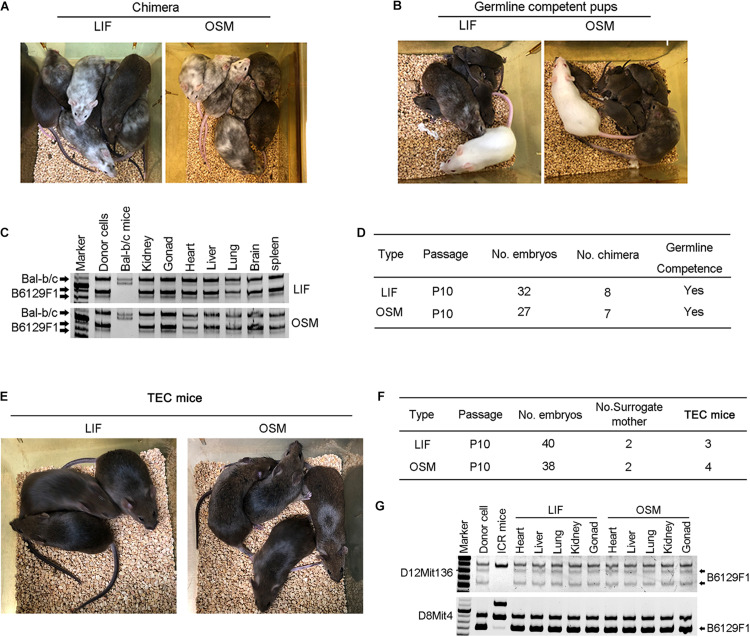
Germline competence chimera and viable tetraploid complementation mice generated from ESCs cultured in OSM medium. **(A,B)** Chimera **(A)** and germline competency **(B)** of mESCs cultured in LIF and OSM medium by 4–8-cell embryo injection. Germline offspring was produced by mating chimeras with albino ICR mice. Albino Balb/c mice served as embryo donors, and pseudo-pregnant albino ICR mice as surrogate mother. **(C)** Genotyping analysis of chimeras by microsatellite primers D12Mit136. **(D)** Summary of 4–8-cell embryo injection of mESCs cultured in LIF and OSM medium. **(E)** Full-ESC mice generated from mESCs cultured in LIF and OSM medium by tetraploid complementation (TEC mice). **(F)** Summary of tetraploid embryo injection of mESCs cultured in LIF and OSM medium. **(G)** Genotyping analysis of TEC mice by microsatellite primers D12Mit136 and D8mit4.

To assess the developmental potential of mESCs cultured in OSM, we performed injections of 4–8-cell embryos. We noted that the mESCs cultured in OSM and LIF exhibited similar efficiency in generating chimeras with germline competence (Figures 3A,B,D). Microsatellite genotyping analysis verified that these mESCs contributed to the generation and development of various tissues including the heart, liver, spleen, lungs, brain, kidneys, and gonads (Figure 3C). To firmly demonstrate their naïve pluripotency, we performed TEC experiment, the most stringent functional test of pluripotency (Eggan et al., 2002). Surrogate female mice naturally delivered TEC pups on approximately day 17.5 of gestation. We found that the mESCs cultured in OSM and LIF achieved similar efficiency in producing TEC pups, and all pups were able to grow healthily into adulthood, being fertile (Figures 3E,F). Microsatellite genotyping analysis confirmed that the examined tissues were molecularly of an ESCs origin (Figure 3G). Both chimera and TEC experiments demonstrated that naïve ESCs cultured in OSM based medium robustly contribute to three germ layers including brain from ectoderm, kidney, heart from mesoderm, and liver, lungs and spleen from endoderm, revealed by microsatellite genotyping analysis (Figures 3C,G). These results verified that OSM was equivalent to LIF in maintaining the naïve pluripotency of ESCs in the TEC assay.

### Transcriptome Profile and Signaling Pathways Regulated by OSM

**FIGURE 4:**
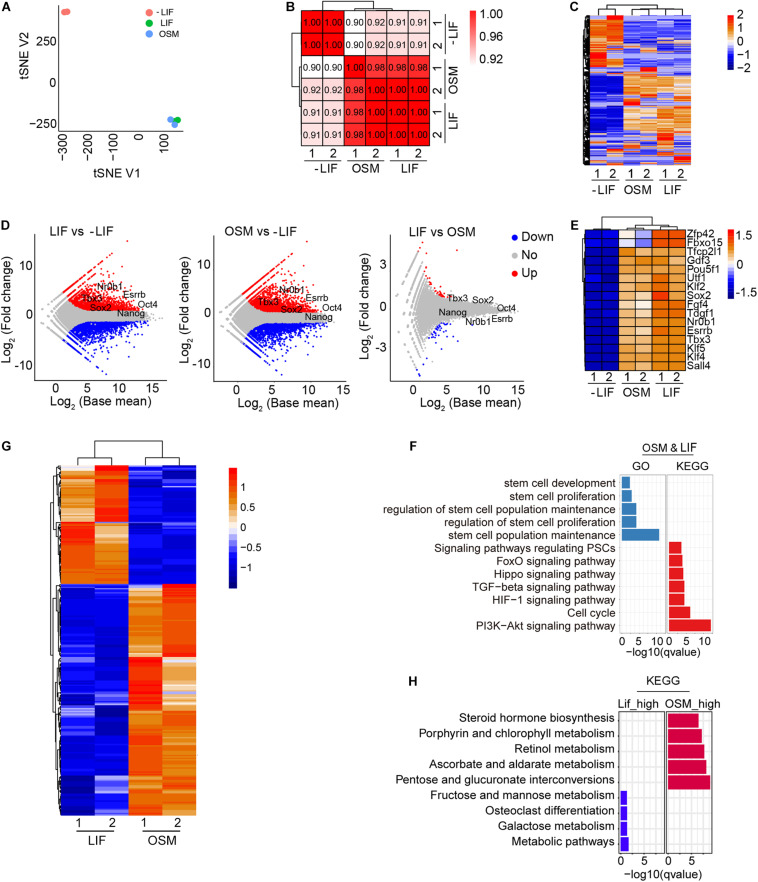
Transcriptome and signal pathways in ESCs cultured in OSM medium. **(A)** tSNE analysis of ESCs cultured in -LIF, LIF, and OSM medium by RNA-seq. **(B)** Pearson’s correlation coefficient graph of mESCs cultured in -LIF, LIF, and OSM medium. The value of 1.0 represents perfect positive correlation and 0 represents no correlation between the two samples. **(C)** Heatmap displaying global gene expression profile of mESCs cultured in -LIF, LIF, and OSM medium. Color key from red to blue represents the relative gene expression level from high to low. **(D)** Scatter-plots showing the differential expression genes in mESCs cultured in -LIF, LIF, and OSM medium. Parallel diagonal lines indicate twofold threshold in expression difference (*P* < 0.05). **(E)** Heatmap of expression profile of pluripotent genes in ESCs cultured in -LIF, LIF, and OSM medium. **(F)** GO and KEGG analysis of genes expressed without differences (fold change < 1.5) in LIF-ESCs and OSM-ESCs. **(G)** Heatmap illustrating differentially expressed genes (DEGs) between mESCs cultured with LIF and with OSM. Two biological replicates were analyzed per group. Genes with ≥0.5-fold expression changes, *P*-value < 0.05 were chosen for heatmap. **(H)** KEGG analysis of upregulated genes in mESCs cultured in OSM medium and those in LIF medium, respectively.

To illustrate the mechanism underlying the maintenance of naïve pluripotency by OSM, we compared the transcriptomes of mESCs cultured in OSM, LIF, and -LIF medium at P10, using RNA-seq analysis. The tSNE and correlation analyses showed that OSM-ESCs clustered closely together with LIF-ESCs, and were obviously separated from -LIF-ESCs (Figures 4A,B). The global gene expression profile revealed substantial similarities between OSM- and LIF-ESCs, compared to -LIF-ESCs (Figures 4C,D). We also observed that pluripotency genes, such as *Oct4*, *Nanog*, *Tbx3*, *Sox2*, *Esrrb*, and *Nr0b1*, were expressed at higher levels in both OSM-ESCs and LIF-ESCs than in -LIF-ESCs (Figures 4D,E), consistent with our immunofluorescence and western blotting data (Figures 1B,C). Moreover, genes regulated DNA methylation and histone methylation and acetylation showed no significant differences between LIF-ESCs and OSM-ESCs except for Dnmt3b and Dnmt3l (Supplementary Figures 1A,B).

Using KEGG pathway and GO term analysis, we found that the genes expressed equivalently in both OSM-ESCs and LIF-ESCs were enriched in pathways including PI3K-Akt signaling (Paling et al., 2004), cell cycle, stem cell maintenance, and proliferation-associated molecular functions, suggesting that these signaling pathways were likely involved in self-renewal and pluripotency maintenance by OSM, as with LIF (Figure 4F). We also examined the differentially expressed genes (DEGs) between OSM-ESCs and LIF-ESCs. Compared to LIF-ESCs, the number of upregulated and downregulated genes in OSM-ESCs were 169 and 87, respectively (Figure 4G and Supplementary Figure 1C). The genes upregulated in OSM-ESCs were enriched in pentose and glucuronate interconversion, ascorbate and aldarate metabolism, and steroid and retinol metabolism (Figure 4H). The genes downregulated in OSM-ESCs were enriched in carbohydrate metabolism, such as fructose and mannose metabolism and galactose metabolism. The role of retinol in supporting the self-renewal of ESCs by elevating the expression of Nanog and Oct4, which are known to be the critical transcription factors for the maintenance of pluripotency of ESCs, has been previously reported (Chen et al., 2007; Chen and Khillan, 2008, 2010). In addition, ascorbate (vitamin C) has been shown to improve the efficiency of generation and quality of induced pluripotent stem cells by modulating histone demethylation (Esteban et al., 2010; Wang et al., 2011; Esteban and Pei, 2012; Chen et al., 2013). To illustrate whether vitamin C (Vc) associated histone modifications might play roles in pluripotency maintenance by OSM, we assessed protein levels of H3K4me3 and H3K27me3 in mESCs cultured in LIF, OSM and LIF medium supplemented with 50 μg/mL Vc. By western blot, level of H3K4me3 did not differ among LIF-ESCs, OSM-ESCs and LIF + Vc-ESCs, but level of H3K27me3 was decreased in OSM-ESCs, and addition of Vc slightly decreased H3K27me3 level of ESCs compared to LIF medium but with no significant differences (Supplementary Figure 2A). Moreover, genomic 5 mC level decreased in OSM-ESCs compared to that of LIF-ESCs by dot blot assay (Supplementary Figure 2B). By real-time qPCR of relative expression levels of selective genes associated with the metabolism, *Ugt1a1* and *Ugt1a6a*, upregulated in OSM-ESCs by RNA-seq, also were expressed at higher levels in OSM-ESCs than in LIF-ESCs (Supplementary Figure 2C). Together, these upregulated metabolic pathways might be involved in the regulation of naïve pluripotency by OSM, but this requires further investigation.

## Discussion

Pluripotent ESCs can differentiate into all cell types in the body and thus have great potential for cell replacement therapy in regenerative medicine, but their culturing requires LIF, a very expensive reagent, to maintain stemness. This expense has limited the number of labs that can participate in stem cell research and has encouraged efforts to find a viable alternative. Several studies have shown that dimethyl sulfoxide (DMSO) (Yi et al., 2020), salvianolic acid B (Liu et al., 2014), and cordycepin (Wang et al., 2020) could maintain the expression of pluripotency markers in mESCs cultured in the absence of LIF; however, the developmental pluripotency of these mESCs was elusive.

That OSM could maintain pluripotency of mESCs has been reported either through morphological criteria (Rose et al., 1994) or through the generation of chimeras (Nichols et al., 1994). The method they used for generation of germline-competent chimeric mice by injecting mESCs into diploid blastocysts provided a valuable but less stringent test of pluripotency (Jaenisch and Young, 2008). Whether OSM could maintain the authentic pluripotency of mESCs through the most stringent tetraploid complementation needed to be addressed. This study reports the first instance of successfully generated all-ESC mice by using the TEC method. In addition, we further examined the effects of OSM on telomere function, 2-cell gene expression, and global gene transcription in naïve mESCs.

Cell metabolism has been shown to be closely related to the pluripotency of ESCs (Gu et al., 2016; Li et al., 2020). We found OSM upregulates ascorbate and retinol metabolic pathways. Future study on these pathways’ function in OSM-maintained pluripotency will expand our understanding of the mechanisms underlying pluripotency. In addition, longer *in vitro* culture is required to confirm the role of OSM in mESC maintenance.

In summary, we demonstrated that OSM can maintain full pluripotency of mESCs in the absence of LIF. This study demonstrates the potential of OSM to completely substitute LIF in mESC cultures, which would be a cost-effective strategy in mESC maintenance.

## Data Availability Statement

The accession number for the RNA-seq data used in this study is GEO: GSE165292 and the other datasets generated during the current study are available from the corresponding author on reasonable request.

## Ethics Statement

The animal study was reviewed and approved by the Institutional Animal Care and Use Committee at Nankai University.

## Author Contributions

XY and CT conducted the experiments and prepared the manuscript. LlL analyzed the RNA-seq data. GF, KJ, HW, and JC conducted part of experiments or provided reagents. LL conceived the project, designed the experiments, wrote, and revised the manuscript. All authors contributed to the article and approved the submitted version.

## Conflict of Interest

The authors declare that the research was conducted in the absence of any commercial or financial relationships that could be construed as a potential conflict of interest.

## References

[B1] Blackburn E. H. Epel E. S. Lin J. (2015). Human telomere biology: a contributory and interactive factor in aging, disease risks, and protection. *Science* 350 1193–1198. 10.1126/science.aab3389 26785477

[B2] Cerulo L. Tagliaferri D. Marotta P. Zoppoli P. Russo F. Mazio C. (2014). Identification of a novel gene signature of ES cells self-renewal fluctuation through system-wide analysis. *PLoS One* 9:e83235. 10.1371/journal.pone.0083235 24392082 PMC3879232

[B3] Chen J. Liu H. Liu J. Qi J. Wei B. Yang J. (2013). H3K9 methylation is a barrier during somatic cell reprogramming into iPSCs. *Nat. Genet.* 45 34–42. 10.1038/ng.2491 23202127

[B4] Chen L. Khillan J. S. (2008). Promotion of feeder-independent self-renewal of embryonic stem cells by retinol (vitamin A). *Stem Cells* 26 1858–1864. 18436859 10.1634/stemcells.2008-0050

[B5] Chen L. Khillan J. S. (2010). A novel signaling by vitamin A/retinol promotes self renewal of mouse embryonic stem cells by activating PI3K/Akt signaling pathway via insulin-like growth factor-1 receptor. *Stem Cells* 28 57–63. 10.1002/stem.251 19890980

[B6] Chen L. Yang M. Dawes J. Khillan J. S. (2007). Suppression of ES cell differentiation by retinol (vitamin A) via the overexpression of Nanog. *Differentiation* 75 682–693. 10.1111/j.1432-0436.2007.00169.x 17451418

[B7] Choi J. Huebner A. J. Clement K. Walsh R. M. Savol A. Lin K. (2017). Prolonged Mek1/2 suppression impairs the developmental potential of embryonic stem cells. *Nature* 548 219–223. 10.1038/nature23274 28746311 PMC5905676

[B8] Eggan K. Rode A. Jentsch I. Samuel C. Hennek T. Tintrup H. (2002). Male and female mice derived from the same embryonic stem cell clone by tetraploid embryo complementation. *Nat. Biotechnol.* 20 455–459. 10.1038/nbt0502-455 11981557

[B9] Esteban M. A. Pei D. (2012). Vitamin C improves the quality of somatic cell reprogramming. *Nat. Genet.* 44 366–367. 10.1038/ng.2222 22456737

[B10] Esteban M. A. Wang T. Qin B. Yang J. Qin D. Cai J. (2010). Vitamin C enhances the generation of mouse and human induced pluripotent stem cells. *Cell Stem Cell* 6 71–79. 10.1016/j.stem.2009.12.001 20036631

[B11] Falco G. Lee S. L. Stanghellini I. Bassey U. C. Hamatani T. Ko M. S. (2007). Zscan4: a novel gene expressed exclusively in late 2-cell embryos and embryonic stem cells. *Dev. Biol.* 307 539–550. 10.1016/j.ydbio.2007.05.003 17553482 PMC1994725

[B12] Gearing D. P. Bruce A. G. (1992). Oncostatin M binds the high-affinity leukemia inhibitory factor receptor. *New Biol.* 4 61–65. 1536831

[B13] Gearing D. P. Comeau M. R. Friend D. J. Gimpel S. D. Thut C. J. McGourty J. (1992). The IL-6 signal transducer, gp130: an oncostatin M receptor and affinity converter for the LIF receptor. *Science* 255 1434–1437. 10.1126/science.1542794 1542794

[B14] Gu W. Gaeta X. Sahakyan A. Chan A. B. Hong C. S. Kim R. (2016). Glycolytic metabolism plays a functional role in regulating human pluripotent stem cell state. *Cell Stem Cell* 19 476–490. 10.1016/j.stem.2016.08.008 27618217 PMC5055460

[B15] Guo R. Ye X. Yang J. Zhou Z. Tian C. Wang H. (2018). Feeders facilitate telomere maintenance and chromosomal stability of embryonic stem cells. *Nat. Commun.* 9:2620. 10.1038/s41467-018-05038-2 29976922 PMC6033898

[B16] Herrera E. Samper E. Martín-Caballero J. Flores J. M. Lee H. W. Blasco M. A. (1999). Disease states associated with telomerase deficiency appear earlier in mice with short telomeres. *EMBO J.* 18 2950–2960. 10.1093/emboj/18.11.2950 10357808 PMC1171377

[B17] Huang J. Deng K. Wu H. Liu Z. Chen Z. Cao S. (2008). Efficient production of mice from embryonic stem cells injected into four- or eight-cell embryos by piezo micromanipulation. *Stem Cells* 26 1883–1890. 10.1634/stemcells.2008-0164 18467666

[B18] Huang J. Wang F. Okuka M. Liu N. Ji G. Ye X. (2011). Association of telomere length with authentic pluripotency of ES/iPS cells. *Cell Res.* 21 779–792. 10.1038/cr.2011.16 21283131 PMC3203670

[B19] Jaenisch R. Young R. (2008). Stem cells, the molecular circuitry of pluripotency and nuclear reprogramming. *Cell* 132 567–582. 10.1016/j.cell.2008.01.015 18295576 PMC4142810

[B20] Kim D. Langmead B. Salzberg S. L. (2015). HISAT: a fast spliced aligner with low memory requirements. *Nat. Methods* 12 357–360. 10.1038/nmeth.3317 25751142 PMC4655817

[B21] Li L. Chen K. Wang T. Wu Y. Xing G. Chen M. (2020). Glis1 facilitates induction of pluripotency via an epigenome-metabolome-epigenome signalling cascade. *Nat. Metab.* 2 882–892. 10.1038/s42255-020-0267-9 32839595

[B22] Liao Y. Smyth G. K. Shi W. (2014). Featurecounts: an efficient general purpose program for assigning sequence reads to genomic features. *Bioinformatics* 30 923–930. 10.1093/bioinformatics/btt656 24227677

[B23] Liu C. H. Shyu W. C. Fu R. H. Huang S. J. Chang C. H. Huang Y. C. (2014). Salvianolic acid B maintained stem cell pluripotency and increased proliferation rate by activating Jak2-Stat3 combined with EGFR-Erk1/2 pathways. *Cell Transplant.* 23 657–668. 10.3727/096368914X678391 24816457

[B24] Liu L. (2017). Linking telomere regulation to stem cell pluripotency. *Trends Genet.* 33 16–33. 10.1016/j.tig.2016.10.007 27889084

[B25] Macfarlan T. S. Gifford W. D. Driscoll S. Lettieri K. Rowe H. M. Bonanomi D. (2012). Embryonic stem cell potency fluctuates with endogenous retrovirus activity. *Nature* 487 57–63. 10.1038/nature11244 22722858 PMC3395470

[B26] Nagy A. Rossant J. Nagy R. Abramow-Newerly W. Roder J. C. (1993). Derivation of completely cell culture-derived mice from early-passage embryonic stem cells. *Proc. Natl. Acad. Sci. U.S.A.* 90 8424–8428. 10.1073/pnas.90.18.8424 8378314 PMC47369

[B27] Nichols J. Chambers I. Smith A. (1994). Derivation of germline competent embryonic stem cells with a combination of interleukin-6 and soluble interleukin-6 receptor. *Exp. Cell Res.* 215 237–239. 10.1006/excr.1994.1338 7957676

[B28] Niwa H. Burdon T. Chambers I. Smith A. (1998). Self-renewal of pluripotent embryonic stem cells is mediated via activation of STAT3. *Genes Dev.* 12 2048–2060. 10.1101/gad.12.13.2048 9649508 PMC316954

[B29] Niwa H. Ogawa K. Shimosato D. Adachi K. (2009). A parallel circuit of LIF signalling pathways maintains pluripotency of mouse ES cells. *Nature* 460 118–122. 10.1038/nature08113 19571885

[B30] Paling N. R. Wheadon H. Bone H. K. Welham M. J. (2004). Regulation of embryonic stem cell self-renewal by phosphoinositide 3-kinase-dependent signaling. *J. Biol. Chem.* 279 48063–48070. 10.1074/jbc.M406467200 15328362

[B31] Raz R. Lee C. K. Cannizzaro L. A. d’Eustachio P. Levy D. E. (1999). Essential role of STAT3 for embryonic stem cell pluripotency. *Proc. Natl. Acad. Sci. U.S.A.* 96 2846–2851. 10.1073/pnas.96.6.2846 10077599 PMC15857

[B32] Rose T. M. Weiford D. M. Gunderson N. L. Bruce A. G. (1994). Oncostatin M (OSM) inhibits the differentiation of pluripotent embryonic stem cells in vitro. *Cytokine* 6 48–54. 10.1016/1043-4666(94)90007-8 8003633

[B33] Wang C. H. Chang C. H. Lin T. L. Fu R. H. Huang Y. C. Chen S. Y. (2020). The novel application of cordycepin in maintaining stem cell pluripotency and increasing iPS cell generation efficiency. *Sci. Rep.* 10:2187. 10.1038/s41598-020-59154-5 32042022 PMC7010772

[B34] Wang T. Chen K. Zeng X. Yang J. Wu Y. Shi X. (2011). The histone demethylases Jhdm1a/1b enhance somatic cell reprogramming in a vitamin-C-dependent manner. *Cell Stem Cell* 9 575–587. 10.1016/j.stem.2011.10.005 22100412

[B35] Yagi M. Kishigami S. Tanaka A. Semi K. Mizutani E. Wakayama S. (2017). Derivation of ground-state female ES cells maintaining gamete-derived DNA methylation. *Nature* 548 224–227. 10.1038/nature23286 28746308

[B36] Yi J. K. Park S. Ha J. J. Kim D. H. Huang H. Park S. J. (2020). Effects of dimethyl sulfoxide on the pluripotency and differentiation capacity of mouse embryonic stem cells. *Cell. Reprogram.* 22 244–253. 10.1089/cell.2020.0006 32936029

[B37] Ying Q. L. Wray J. Nichols J. Batlle-Morera L. Doble B. Woodgett J. (2008). The ground state of embryonic stem cell self-renewal. *Nature* 453 519–523. 10.1038/nature06968 18497825 PMC5328678

[B38] Yu G. Wang L. G. Han Y. He Q. Y. (2012). clusterProfiler: an R package for comparing biological themes among gene clusters. *OMICS* 16 284–287. 10.1089/omi.2011.0118 22455463 PMC3339379

[B39] Zalzman M. Falco G. Sharova L. V. Nishiyama A. Thomas M. Lee S. L. (2010). Zscan4 regulates telomere elongation and genomic stability in ES cells. *Nature* 464 858–863. 10.1038/nature08882 20336070 PMC2851843

[B40] Zarling J. M. Shoyab M. Marquardt H. Hanson M. B. Lioubin M. N. Todaro G. J. (1986). Oncostatin M: a growth regulator produced by differentiated histiocytic lymphoma cells. *Proc. Natl. Acad. Sci. U.S.A.* 83 9739–9743. 10.1073/pnas.83.24.9739 3540948 PMC387216

