# Supplementary material for: Oncostatin M Maintains Naïve Pluripotency of mESCs by Tetraploid Embryo Complementation (TEC) Assay
Source: Front Cell Dev Biol. 2021 May 26;9:675411. doi: 10.3389/fcell.2021.675411 (PMC8189179; doi:10.3389/fcell.2021.675411)
Supplement: Supplementary file 1 [file Data_Sheet_1.pdf]

Supplementary Material

Supplementary Figures

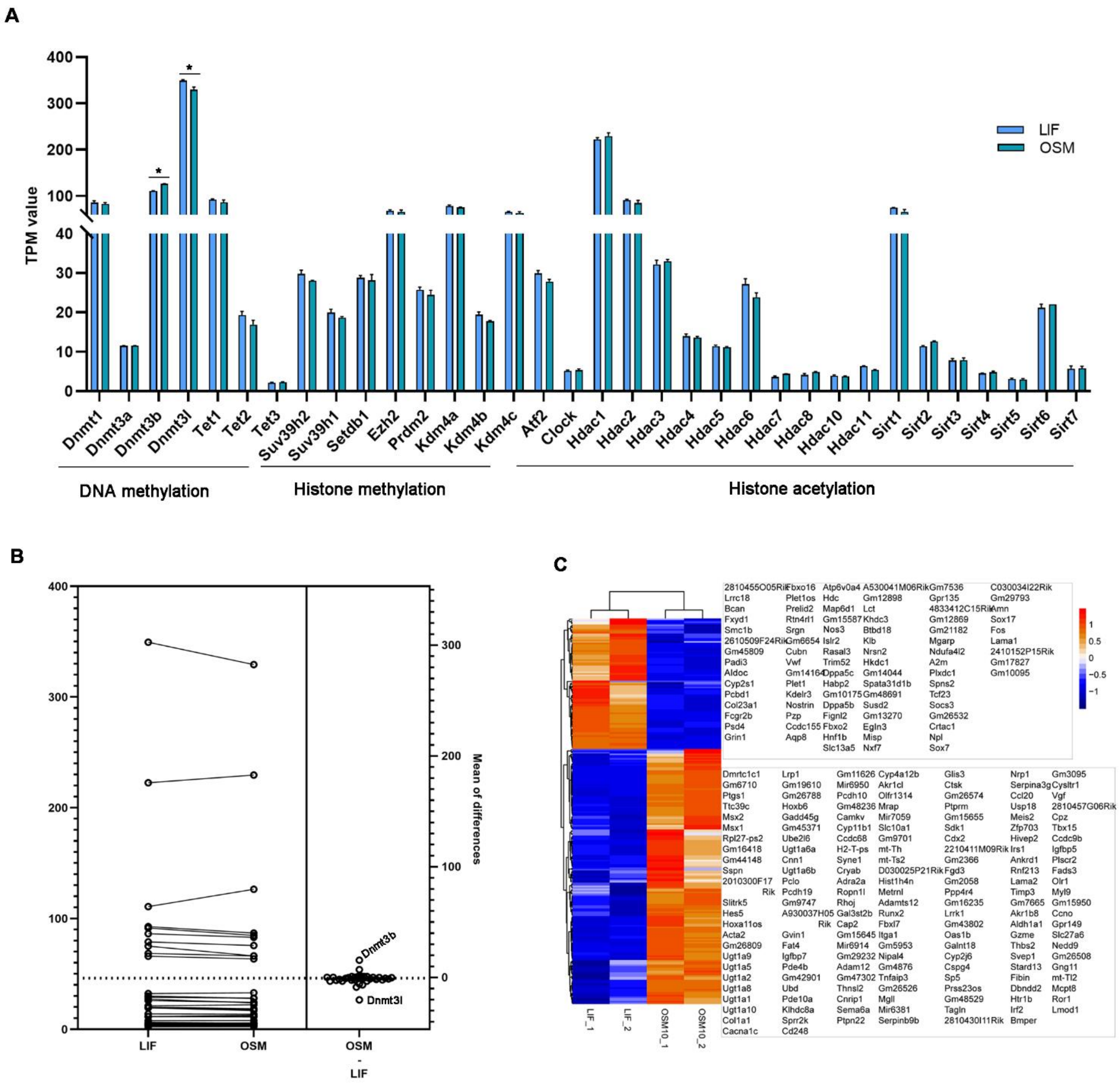

Figure S1. Epigenetic related gene expression in mESCs cultured in LIF and in OSM medium by RNA-seq.

(A) Expression of genes for DNA methylation, histone methylation and acetylation by RNA-seq analysis.
(B) Estimation Plot shows mean of differences of expression levels of genes (listed in A) between mESCs cultured in LIF or OSM medium.
(C) Heatmap illustrating differentially expressed genes (DEGs) between mESCs cultured with LIF and with OSM. Two biological replicates were analyzed per group. Genes with ⩾1.5-fold expression changes, P value < 0.05 were chosen for heatmap.

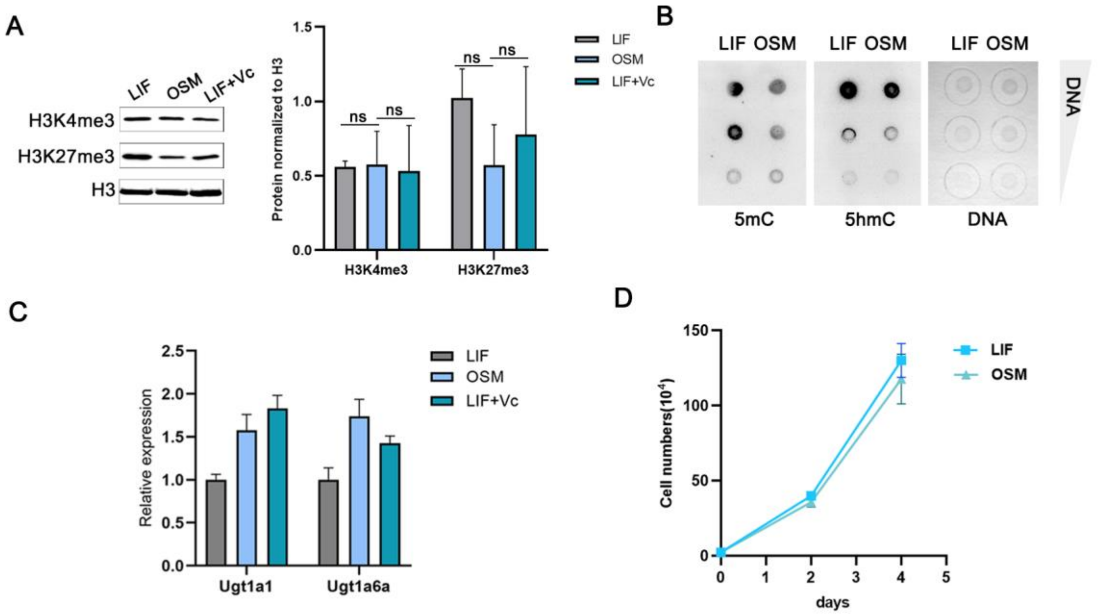

Figure S2. Histone modification and DNA methylation changed in mESCs cultured in OSM medium and LIF medium supplemented with vitamin C (Vc).
(A) Western blot analysis of H3K4me3 and H3K27me3 in mESCs cultured in LIF, OSM and LIF+Vc medium. Concentration of Vc (2-Phospho-L-ascorbic acid trisodium salt, Sigma, 49752) was at 50μg/mL. Relative quantification of H3K4me3 and H3K27me3 expression levels was obtained from two independent experiments.
(B) Dot blot analysis of the genomic 5mC and 5hmC levels in mESCs cultured in LIF or OSM medium.
(C) Relative expression by real-time qPCR of uridine diphosphoglucuronosyl transferases family gene Ugt1a1 and Ugt1a6a, in ESCs cultured in LIF, OSM and LIF+Vc medium.
(D) Cell proliferation of ESCs cultured in LIF or OSM.

Supplementary Tables

Table S1. Primers for real-time qPCR analysis.

Genes	Forward	Reverse
Tert	ACTGGTGGAGATCATCTTTCTGGG	ACCTGAGGAGTCTGACATATTGGC
Terc	CATTAGCTGTGGGTTCTGGTCT	TCCTGCGCTGACGTTTGTTT
Zscan4	AAATGCCTTATGTCTGTTCCCTATG	TGTGGTAATTCCTCAGGTGACGAT
Tcstv1	TGAACCCTGATGCCTGCTAAGACT	AGATGGCTGCAAAGACACAACTGC
Tcstv3	AGAAAGGGCTGGAACTTGTGACCT	AAAGCTCTTTGAAGCCATGCCCAG

Ugt1a1	AGCCTATGTCAACGCCTCTG	GGTCTAGTTCCGGTGTAGCG
Ugt1a6a	GGTCCTAGTGCCAGAAGTCA	GAGGCACCGGGAAGAAAGTG
Gapdh	TCAACAGCAACTCCCACTCTTCCA	ACCACCCTGTTGCTGTAGCCGTAT

Table S2. Primers for PCR in microsatellite genotyping

	Forward	Reverse
D12Mit136	TTAATTTTGAGTGGGTTTGGC	TTG CTACATGTACACTGATCTCCA
D8Mit4	GGCTGCAATGCGGTTCCGTAGA	TGAAGAGGACGCTTTGGATGAT

Supplementary experimental procedure

Dot blot

For dot blot, DNA were denatured at 99°C for 5 min then spotted onto nylon membranes (RPN2020B; GE Healthcare). After UV cross-linking membranes were blocked 30min with 5% non-fat milk in PBS-T, then incubated for one hour with 5mC (Active Motif, 39649) or 5hmC (Active Motif, 39769) antibodies at room temperature, followed by incubation for 30 min with HRP conjugated goat anti-Rabbit IgG (Abcam, ab136817) and goat anti-mouse IgG Abcam, ab136815), and visualized using the Immobilon Western HRP Substrate (WBKLS0500; Millipore).
